# Draft Genome Sequence of Pandrug-Resistant Pseudomonas aeruginosa SPA03, Isolated from a Patient with Benign Prostatic Hyperplasia

**DOI:** 10.1128/MRA.00336-21

**Published:** 2021-06-03

**Authors:** Chanakya Pachi Pulusu, Balaram Khamari, Manmath Lama, Arun Sai Kumar Peketi, Prakash Kumar, Valakunja Nagaraja, Eswarappa Pradeep Bulagonda

**Affiliations:** a Department of Biosciences, Sri Sathya Sai Institute of Higher Learning, Puttaparthi, India; b Department of Microbiology, Sri Sathya Sai Institute of Higher Medical Sciences, Prasanthigram, India; c Department of Microbiology and Cell Biology, Indian Institute of Science, Bangalore, India; d Jawaharlal Nehru Centre for Advanced Scientific Research, Bangalore, India; University of Arizona

## Abstract

The draft genome of pandrug-resistant Pseudomonas aeruginosa strain SPA03, which belongs to global high-risk sequence type 357 (ST357) and was isolated from a patient with benign prostatic hyperplasia, is presented in this report. The genome assembly was generated by combining short-read Illumina HiSeq-X Ten and long-read Oxford Nanopore Technologies MinION sequence data using the Unicycler assembler.

## ANNOUNCEMENT

Pseudomonas aeruginosa, a member of the WHO priority pathogen list (1), is an opportunistic human pathogen that infects vulnerable patients (2, 3). Urinary tract infections (UTIs) due to P. aeruginosa are often associated with increased morbidity and mortality rates (4). Although several genomes of P. aeruginosa are available in the GenBank database, the emergence of pandrug-resistant (PDR) variants belonging to global high-risk clone sequence type 357 (ST357) warrants the sequencing of additional PDR clinical isolates.

Here, we present a draft genome sequence of PDR P. aeruginosa strain SPA03, which was isolated from the catheter urine of a 77-year-old male patient with benign prostatic hyperplasia. Ethical approval was obtained from the Sri Sathya Sai Institute of Higher Learning Institutional Ethics Committee (approval number SSSIHL/IEC/PSN/BS/2014/03). A clean urine aspirate sample from the urinary catheter was cultured on sheep blood agar (HiMedia) for 24 h at 37°C. The isolate was identified as P. aeruginosa with matrix-assisted laser desorption ionization–time of flight mass spectrometry (MALDI-TOF MS) (bioMérieux), and this was confirmed by ribosomal multilocus sequence typing (MLST) (5). The strain was found to be PDR, exhibiting resistance to all tested antibiotics (Table 1), by Vitek 2 (AST N281 card).

**TABLE 1:** Antimicrobial susceptibility profile of P. aeruginosa SPA03

Antimicrobial	MIC (μg/ml)^*a*^	Resistance profile
Ticarcillin-clavulanic acid	≥128	R^*b*^
Piperacillin-tazobactam	≥128	R
Ceftazidime	≥64	R
Cefoperazone-sulbactam	≥64	R
Cefepime	≥64	R
Doripenem	≥8	R
Imipenem	≥16	R
Meropenem	≥16	R
Amikacin	≥64	R
Gentamicin	≥16	R
Ciprofloxacin	≥4	R
Levofloxacin	≥8	R
Minocycline	≥16	R
Tigecycline	≥8	R
Colistin	4	R
Trimethoprim-sulfamethoxazole	≥320	R

a Antibiotic susceptibility testing was performed using the Vitek 2 system according to Clinical and Laboratory Standards Institute (CLSI) guidelines (15).

b R, resistant.

Genomic DNA extracted with the Macherey-Nagel NucleoSpin DNA extraction kit from the culture of a single colony grown overnight in LB broth at 37°C was used for the preparation of an Illumina (paired-end) sequencing library and a Nanopore sequencing library (prepared using sheared DNA) with the NEBNext Ultra II DNA library preparation kit (E7645S) and the Nanopore ligation sequencing kit (SQK-LSK109), respectively. Quality and quantity were assessed using a Qubit 2 fluorometer and an Agilent Bioanalyzer DNA 1000 kit. An Illumina HiSeq-X Ten system was used to sequence the libraries, and demultiplexing was performed using bcl2fastq v2.2. A total of 4,722,619 read pairs were generated using 2 × 150-bp chemistry. Adapter sequences were removed using fastp v0.20.1 (6). Nanopore sequencing was performed with a FLO-MIN106 flow cell on a MinION device (Oxford Nanopore Technologies). Base calling and demultiplexing were performed using Albacore v2.0.1, and adapter sequences were removed using Porechop v0.2.4 with default settings. The MinION sequencing run generated 90,337 reads, with an average length of 4,961 bp and a mean quality score of 8.9 (NanoStat) (7). The processed reads from Nanopore and Illumina sequencing were used to generate a hybrid *de novo* assembly using Unicycler v0.4.8 (8). Final assembly quality was assessed with QUAST (9). The SPA03 genome was annotated using the NCBI Prokaryotic Genome Annotation Pipeline (PGAP) v5.0 (10). Default parameters were used for all software unless otherwise specified.

The hybrid genome assembly consists of 9 contigs, with a total length of 6,829,266 bp (*N*_50_, 5,225,585 bp; *N*_75_, 5,225,585 bp), a GC content of 65.9%, and genome coverage of 116.13×. A total of 6,307 genes were predicted by the NCBI PGAP, including 6,153 protein-coding sequences, 9 rRNAs, 62 tRNAs, 4 noncoding RNAs, and 79 pseudogenes. MLST analysis by MLST v2.0 (11) and serotyping by Past v1.0 (12) identified that the study genome belongs to ST357 and serogroup O11. ResFinder v4.1 (13) and CARD v3.1.1 (14) analyses revealed the presence of antibiotic resistance genes for aminoglycosides, β-lactams, fluoroquinolones, fosfomycin, chloramphenicol, rifampin, sulfonamides, and tetracyclines, along with multiple efflux pump genes.

In summary, we report the draft genome of PDR P. aeruginosa SPA03, which belongs to high-risk clone ST357. Early identification of such pathogens could help in investigating outbreaks and controlling infections. The whole-genome sequencing data for PDR clinical isolates would serve as a useful reference for genome assemblies and comparator strains for further research.

### Data availability.

The whole-genome sequence data and raw sequence data for P. aeruginosa strain SPA03 are available at NCBI under BioProject PRJNA689041, GenBank accession number GCA_016595165.1, and Sequence Read Archive (SRA) accession numbers SRX10461636 (Illumina raw sequence data) and SRX10461637 (MinION raw sequence data).
